# International standards and good practice guidelines in traditional, complementary and integrative medicine: a scoping review

**DOI:** 10.3389/fphar.2026.1742400

**Published:** 2026-04-09

**Authors:** Xuanbin Wang, Hongliang Li, Shun Jiang, Nian Yang, Dongpeng Wang, Hongxi Xu, Nicola Robinson, Lie-Fen Shyur, Michael Heinrich, Mei Wang, Monique S. J. Simmonds, Linda Zhong, Fatiha Brahmi, Thomas Efferth, Salvius Amuri Bakari, Clara Bik-San Lau, Wendy Wong, Rudolf Bauer, Pierre Duez, Qihe Xu

**Affiliations:** 1 Laboratory of Chinese Herbal Pharmacology, Department of Pharmacy, Renmin Hospital, Institute of Biomedicine Research, Hubei Key Laboratory of Wudang Local Chinese Medicine Research, Hubei University of Medicine, Shiyan, China; 2 The First Affiliated Hospital (Shenzhen People’s Hospital), Southern University of Science and Technology, Shenzhen, China; 3 School of Pharmacy, Shanghai University of Traditional Chinese Medicine, Shanghai, China; 4 London South Bank University, London, United Kingdom; 5 Centre of Evidence Based Medicine, Beijing University of Chinese Medicine, Beijing, China; 6 Agricultural Biotechnology Research Center, Academia Sinica, Taipei, Taiwan; 7 Research Group 'Pharmacognosy and Phytotherapy’, UCL School of Pharmacy, University College London, London, United Kingdom; 8 Department of Pharmaceutical Sciences and Chinese Medicine Resources, Chinese Medicine Research Center, College of Chinese Medicine, China Medical University, Taichung, Taiwan; 9 Naturalis Biodiversity Center, and SU BioMedicine B.V., Leiden, Netherlands; 10 Royal Botanic Gardens Kew, London, United Kingdom; 11 Biomedical Sciences and Chinese Medicine, School of Biological Sciences, Nanyang Technological University, Singapore, Singapore; 12 NTU Chinese Medicine Clinic, Nanyang Technological University, Singapore, Singapore; 13 Laboratory of Biomathematics, Biochemistry, Biophysics and Scientometry, Faculty of Natural and Life Sciences, University of Bejaia, Bejaia, Algeria; 14 Department of Pharmaceutical Biology, Institute of Pharmaceutical and Biomedical Sciences, Johannes Gutenberg University, Mainz, Germany; 15 Laboratory of Pharmacognosy, Faculty of Pharmaceutical Sciences, University of Lubumbashi, Lubumbashi, Democratic Republic of Congo; 16 Department of Pharmacology and Pharmacy, and School of Chinese Medicine, LKS Faculty of Medicine, The University of Hong Kong, Hong Kong SAR, China; 17 Jockey Club of School of Public Health and Primary Care, Chinese University of Hong Kong, Hong Kong SAR, China; 18 Institute of Pharmaceutical Sciences, University of Graz, Graz, Austria; 19 Unit of Therapeutic Chemistry and Pharmacognosy, University of Mons (UMONS), Mons, Belgium; 20 Department of Inflammation Biology, School of Immunology and Microbial Sciences, Faculty of Life Sciences and Medicine, King’s College London, London, United Kingdom; 21 King’s Centre for Integrative Chinese Medicine, James Black Centre, King’s College London, London, United Kingdom

**Keywords:** complementary medicine, integrative medicine, international good practice guidelines, international standards, scoping review, traditional medicine

## Abstract

**Objectives:**

To map the development of international standards (IS) and international good practice guidelines (IGPG) across the field of traditional, complementary and integrative medicine (TCIM) and establish a comprehensive repository.

**Methods:**

A systematic search was conducted using PubMed, Web of Science, EMBASE, ProQuest, and the Cochrane Library, as well as relevant websites, with the assistance of artificial intelligence tools. This search combined MeSH terms and keywords, and was further supplemented by non-systematic human expert input, covering the period from January 2000 to April 2025. Duplicates were removed and all records were screened based on pre-defined criteria for TCIM-relevant IS/IGPG and TCIM- and IS/IGPG-related systematic reviews, implementation documents and commentaries.

**Findings:**

2026 records met inclusion criteria: (a) TCIM-relevant IS/IGPG documents (n = 1,624); and (b) TCIM- and IS/IGPG-related secondary documents (systematic reviews, perspectives and commentaries, n = 402). These IS/IGPG were produced by 33 international organisations and consortia, broadly applicable to TCIM or specific to a particular TCIM modality. Our data showed acceleration in IS/IGPG production over the past two decades. An analysis of the secondary literature provided a broad overview of the disease spectrum and the application of IS/IGPG in TCIM studies.

**Conclusion:**

A comprehensive repository for TCIM-related IS/IGPG has been established. These IS/IGPG can be expected to play important roles for an efficient implementation of the World Health Organization Traditional Medicine Strategy 2025–2034. Future work should focus on disseminating, implementing and harmonising these IS/IGPG, evaluating their effectiveness and refining them, while promoting global parity in access, implementation and coverage.

**Study Registration:**

The Open Science Framework (https://doi.org/10.17605/OSF.IO/H8UFM).

## 1 Introduction

Ensuring universal access to safe, effective, and people-centred traditional, complementary and integrative medicine (TCIM) constitutes a core objective of the World Health Organization (WHO) Traditional Medicine Strategy 2025–2034 and is an essential long-term aim of research in the field of ethnopharmacolgy. TCIM encompasses multiple healing systems practised alongside or in combination with modern conventional medicine. This includes *traditional medicine*, *i.e.*, historically and culturally rooted health systems that pre-date modern conventional medicine and emphasise nature-based remedies and integrative, personalised care to restore balance between mind, body and environment; *complementary medicine*, formerly known as *complementary and alternative medicine*, which refers to health practices used alongside a country’s conventional care to support health and wellness; and *integrative medicine*, an interdisciplinary, evidence-based approach combining traditional and/or complementary medical knowledge, skills and practices with conventional care (World Health Organization, 2025a).

Traditional medicine comprises diverse modalities, as exemplified by traditional Chinese medicine (TCM), which includes Chinese herbal medicine, acupuncture, moxibustion, cupping, tuina, taiji (tai chi), guasha and other specialised diagnostic and interventional technologies. Similarly, traditional Indian medicine, which encompasses a range of traditional practices, *e.g.*, ayurveda, unani, siddha, naturopathy and yoga, and European traditional herbal medicines, also have substantial global reach and impact. Furthermore, there are numerous other regional and cultural traditions in Africa, America, Arabic and Middle-East countries, Australia, etc., which are less known globally but warrant further exploration.

TCIM has substantial global reach and influence. Approximately 80% countries officially recognise the use of acupuncture (World Health Organization, 2013), while an estimated 80% of the population in sub-Saharan Africa depends on traditional herbal medicine for primary healthcare (Kahumba et al., 2015). In China, TCM outpatient visits considerably rose, from 146 million in 2002 to 1.54 billion in 2023, with 99.6% of community clinics offering TCM services by 2023 (National Health Commission of the People’s Republic of China, 2003; 2024). Likewise, in the United States, reported adult use of TCIM rose from 19.2% in 2002 to 36.7% in 2022 (Nahin et al., 2024). In response to this growing prominence of TCIM, the new WHO Strategy outlines four key objectives: strengthening the evidence base, establishing robust regulation, acknowledging and integrating recognised practitioners and safe and effective practices/products into national health systems, and promoting cross-sectoral value and community empowerment (Burki, 2025; World Health Organization, 2025a). Achieving these goals requires rigorous research that not only respects relevant cultural contexts, but also is underpinned by international standards (IS), which are formal, often certifiable, established documents, developed and published by internationally recognised standards bodies through a process of consensus among its member countries, and international good practice guidelines (IGPG), which are recommended approaches or processes recognised as being superior to alternatives, representing the collective understanding and experience of a field (Von Schoen-Angerer et al., 2023).

In the intrinsically diverse and structurally complex field of TCIM, the use of preparations and metabolites derived from natural sources is common, constituting the main focus of this systematic review, which also covers other TCIM modalities, such as acupuncture and other physical medical interventions. Relevant IS/IGPG documents are produced by multiple international stakeholders and remain dispersed across numerous repositories. To address this fragmentation and improve accessibility, this scoping review was designed primarily to establish a unified repository by systematically mapping the global landscape of IS/IGPG development; a secondary aim was to compile TCIM- and IS/IGPG-related secondary documents, *e.g.*, systematic reviews, perspectives and commentaries, as a proxy for IS/IGPG use and clinical evidence across the TCIM field.

## 2 Methods

### 2.1 Research design

This scoping review was conducted in line with the PRISMA-ScR guidelines (Tricco et al., 2018) and structured using an ICC framework, encompassing three domains, *i.e., Information, Concepts* and *Context*, as adapted from a reported PCC (*Population, Concept* and *Context*) framework (Chipps et al., 2025).

- Information: TCIM-related IS/IGPG.
- Concepts: Development and dissemination of IS/IGPG.
- Context: Progress, barriers, challenges and outlooks in IS/IGPG development and dissemination.

### 2.2 Search strategy

The strategies for systematic searches across five databases, PubMed, Web of Science, EMBASE, ProQuest, Cochrane Library (Supplementary Table S1) were supplemented by artificial intelligence (AI)-driven grey literature retrieval from the websites of relevant international organisations and industrial alliances (Supplementary Table S2), using a large language model (LLM), Claude Opus 4 (Anthropic, San Francisco, CA, United States). Data retrieved by LLM were extracted and reported following the TITAN Guidelines 2025 (Agha et al., 2025). To avoid artefacts brought about by LLM, all records were systematically evaluated manually against predefined exclusion and inclusion criteria and supplemented by invited experts from international organisations and relevant professional settings.

#### 2.2.1 Inclusion criteria

- Documents relevant to TCIM (examples in Supplementary Table S3);
- Document type - standards, guidelines, benchmarks, frameworks, strategies or white papers;
- Publications on implementation, application, promotion, and enforcement of IS and IGPG;
- Secondary documents (e.g., systematic reviews, scoping reviews, meta-analyses, Cochrane library reports) relevant to clinical evidence of TCIM obtained from studies applying IS and IGPG;
- Date of publications (01/2000 to 04/2025); and
- No language restriction was applied.

#### 2.2.2 Exclusion criteria

- Documents irrelevant to TCIM or IS/IGPG;
- Primary research studies or case reports; and
- Unfinished or draft documents, non-peer-reviewed preprints, and general news coverage.

### 2.3 Data extraction and analysis

At least two authors independently reviewed extracted data and assessed the quality of documents. Duplicates were removed manually and further supplemented by non-systematic searches and input by human experts. Any disagreements were resolved by consensus, yielding a fully authenticated corpus of documents with consistently high inter-rater agreement. The literature data were organised using Microsoft Excel and EndNote 21 (Clarivate, Philadelphia, PA, United States).

## 3 Results

### 3.1 Flowchart of literature screening

**FIGURE 1:**
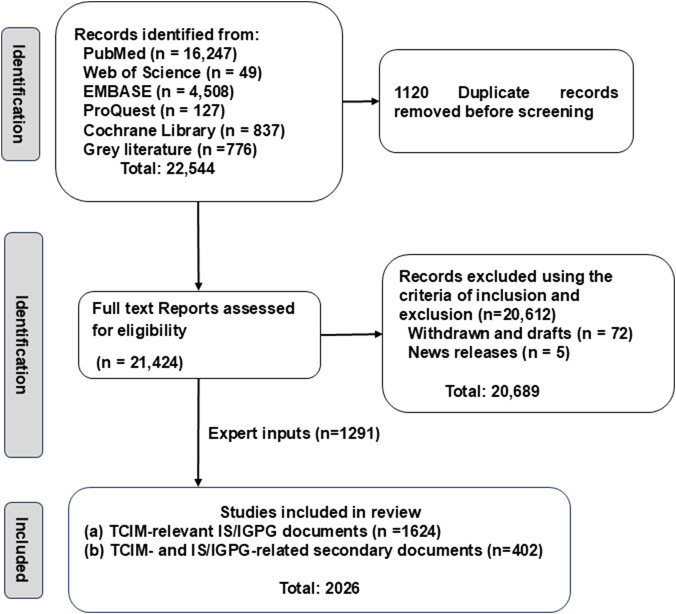
Flowchart of data extraction on the international standards (IS) and international good practice guidelines (IGPG) for TCIM in 2000–2025.

The data extraction process followed the PRISMA 2020 flowchart (Figure 1). Initial searches identified 22,544 records. After exclusion of 1,120 duplicates, eligibility assessment for full-text retrieval was conducted against predefined inclusion and exclusion criteria, which excluded withdrawn manuscripts, draft documents and general news coverage (n = 20,689). Through team deliberation and author consensus, 735 records were retained for analysis and supplemented by 1,291 non-systematic inputs by experts. A total of 2026 records were categorised as follows.

- Category (a) international standards and guidelines (n = 1,624, Supplementary Table S4) and
- Category (b) systematic reviews, perspectives and commentary documents (n = 402, cited in Supplementary Table S5).

### 3.2 IS/IGPG relevant to TCIM

**FIGURE 2:**
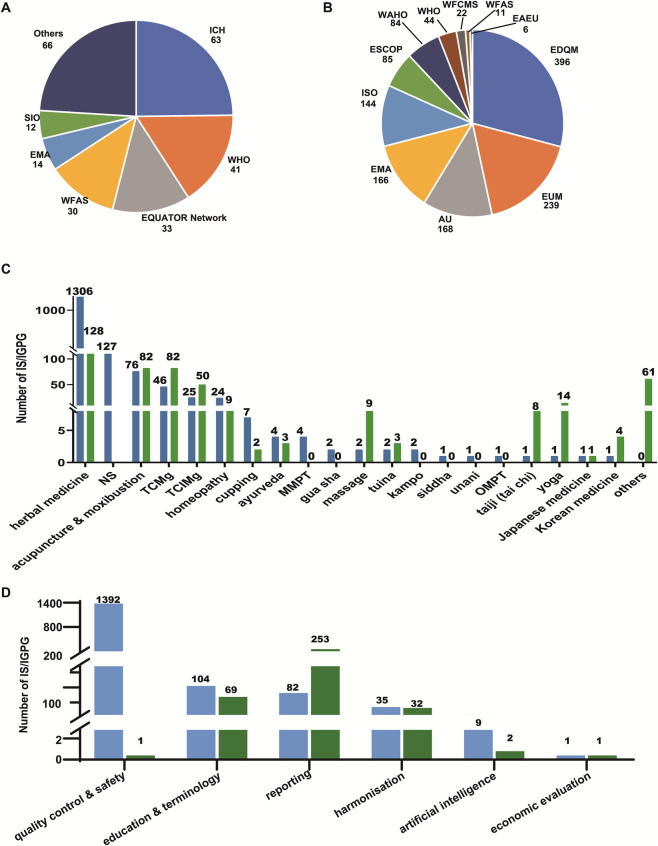
Global landscape of the development and use of IS and IGPG in 2000–2025. **(A)** Proportions of contribution to the IGPG included in this study by international organisations and consortia. “Others”: databases, 20; CIOMS, 6; GIN, 5; IFOMPT, 5; PAHO, 3; SAR, 3; AAPB, 2; EUSOMA, 2; HRC, 2; WFCMS, 2; AOAC, 1; CA, 1; COPE,1; DTx, 1; GA, 1; GHC, 1; HIMSS, 1; ICMJE, 1; LAP, 1; PROREUS, 1; TRAFFIC, 1. **(B)** Proportions of contribution to the IS included in this study by international organisations and consortia. **(C)** TCIM modalities covered by the included IS/IGPG; blue bars: IS/IGPG documents; green bars: TCIM- and IS/IGPG-related secondary documents. **(D)** Themes of the included IS/IGPG; blue bars: IS/IGPG documents; green bars: TCIM- and IS/IGPG-related secondary documents. AAPB, Association for Applied Psychophysiology and Biofeedback; AOAC, Association of Official Agricultural Chemists International; AU, African Union; CA, Comunidad Andina (*La Comunidad Andina*); CIOMS, Council for International Organizations of Medical Sciences; COPE, Committee on Publication Ethics guidelines; DTx, Digital Therapeutics Alliance; EAEU, Eurasian Economic Union; EMA/HMPC, European Medicines Agency/Committee on Herbal Medicinal Products; EDQM, European Directorate for the Quality of Medicines and Healthcare; EQUATOR Network, Enhancing the QUAlity and Transparency Of health Research; ESCOP, European Scientific Cooperative on Phytotherapy; EUM, Estados Unidos Mexicanos; EUSOMA, European Society of Breast Cancer Specialists; GA, Society for Medicinal Plant and Natural Product Research; GBIF, Global Biodiversity Information Facility; GCRSR, Global Coalition for Regulatory Science Research; GHC/GCC, The Gulf Health Council/ The Gulf Cooperation Council; GIN, Guidelines International Network; HIMSS, Healthcare Information and Management Systems Society; HL7 International, Health Level Seven International; HRC, The Pacific Health Research Committee and the Health Research Council of New Zealand; ICH, International Council for Harmonization of Technical Requirements for Pharmaceuticals for Human Use; ICMJE, International Federation of Orthopaedic Manipulative Physical Therapist; IGPG, international good practice guidelines; IS, international standards; ISCMR, International Society for Complementary Medicine Research; ISE, International Society for Ethnopharmacology; ISO, International Organisation for Standardisation; LAP, Latin American Parliament; MMPT, manual and musculoskeletal physical therapies; NS: not specific, but nonetheless highly relevant, to TCIM; OMPT, orthopaedic manipulative physical therapies; PAHO, Pan American Health Organization; SAR, Society for Acupuncture Research; SIO, Society for Integrative Oncology; SIOP, International Society of Paediatric Oncology; TCIMg: General IS/IGPG in the broad field of traditional, complementary and integrative medicine, TCMg: General IS/IGPG in the field of traditional Chinese medicine; TRAFFIC, the long-term vision of the Kunming-Montreal Global Biodiversity Framework; WAHO, West African Health Organization; WFAS, World Federation of Acupuncture-moxibustion Societies; WFCMS, World Federation of Chinese Medicine Societies; WHO, World Health Organization.

Of the 1,624 records in Category (a) (Supplementary Table S4), including both IS (n = 1,365) and IGPG (n = 259), 22 were retrieved from the five bibliographic databases, with the remaining 1,602 sourced from online grey literature deposited at the websites of 33 international organisations and agencies, such as European Directorate for the Quality of Medicines and Healthcare (EDQM), Estados Unidos Mexicanos (EUM), African Union (AU), European Medicines Agency (EMA), International Organization for Standardization (ISO), the European Scientific Cooperative on Phytotherapy (ESCOP), the International Council for Harmonisation of Technical Requirements for Pharmaceuticals for Human Use (ICH), WHO, and the Enhancing the QUAlity and Transparency Of health Research (EQUATOR) Network (Figures 2A,B). These IS/IGPG were either broadly specific to TCIM, specific to a TCIM modality, or relevant but non-specific to TCIM (Figure 2C). The modalities of TCIM covered by IS/IGPG included acupuncture and moxibustion, herbal medicine, cupping, and some other complementary medical techniques derived from Chinese, Indian, Korean, Japanese or other medical traditions or relatively modern medical systems. These IS/IGPG were particularly focused on quality control and safety, education and terminology, reporting, harmonisation, AI, and economic evaluation (Figure 2D). Analysis of 5-year publication intervals over the past 25 years revealed a pronounced acceleration in IS/IGPG production in the past two decades, with outputs rising from 58 (2000–2005), 279 (2006–2010) and 279 (2011–2015) to 431 (2016–2020) and 562 (2021–2025) documents.

### 3.3 Analysis of TCIM- and IS/IGPG-related secondary documents

Among 402 records in Category (b), 215 were retrieved from PubMed, 78 from EMBASE, 27 from ProQuest and 77 from Cochrane, while five were retrieved from grey literature. All Web of Science records were duplicates of those in PubMed and, therefore, excluded. These records included a total of 20,802 studies that involved TCIM and cited IS/IGPG, allowing for an analysis of TCIM modalities, IS/IGPG types, authorship and their countries, evidence levels and quality, both individually and collectively (Supplementary Table S5). The data also provided a cross-sectional overview of the disease spectrum in TCIM studies that applied or referred to IS/IGPG. When categorised according to the WHO International Classification of Diseases, 11th Revision (ICD-11), the analysis identified 103 specific diseases, a significant expansion from the 23 general disorder types noted in the pre-2002 period, as highlighted by the WHO Traditional Medicine Strategy 2002–2005 (World Health Organization, 2002a). These results yielded a glimpse of progress in the past two decades through a nuanced comparison (Supplementary Table S6).

## 4 Discussion

### 4.1 IS/IGPG for evidence-based TCIM

Over the past two decades, there has been a remarkable acceleration in the development of IGPG and IS (Figures 2A,B; Supplementary Table S4), including those specific to TCIM, to a particular TCIM modality, or important for, although not specific to, TCIM. Regarding TCIM modalities, the highest number of modality-specific IS/IGPG concern herbal medicine, acupuncture and moxibustion, homeopathy and cupping, (Figure 2C), in keeping with the largest volumes of systematic review evidence for these modalities between 2018 and 2022, as documented by the WHO (World Health Organization, 2023b). The IS/IGPG compiled in this scoping review should be integrated with more general guidelines to ensure comprehensive, context-dependent coverage for both conventional medicine and TCIM. It’s also important to recognise a dynamic nature of IS/IGPG. As any published IS/IGPG is likely to be regularly updated, it is essential to double-check and identify the most updated guidelines and TCIM-specific extensions while designing and reporting any work. For example, with the recent publication of the updated SPIRIT 2025 and CONSORT 2025 guidelines (Hopewell et al., 2025; Hróbjartsson et al., 2025), any randomised control trial protocols and reports in the field of TCIM should refer to these revised guidelines in addition to any updated specific guidelines. Just as this manuscript went into final production, the Second Edition of the African Herbal Pharmacopoeia was published. This volume consolidates scientific and ethnobotanical knowledge on 30 of the most significant medicinal plants in Africa (https://www.routledge.com/African-Herbal-Pharmacopoeia/Katerere-Brendler-Feiter-Mahomoodally-Phillips/p/book/9780815374244). As time progresses, we can expect further IS/IGPG publications to emerge in the coming years.

IS/IGPG are crucial for the modernisation of traditional medicine, an effort to bring ancient traditional practices into line with modern scientific standards (Xu et al., 2013). However, modernisation must not come at the expense of traditional medicine’s core values, notably its integrative approach and its foundation in patients-centred, personalised, syndrome-based differential diagnosis. Obviously, IS/IGPG should be applied and further refined with respect to these important concepts. For example, *(i)* TCIM’s emphasis of patients-reported outcomes need to be acknowledged and included in clinical practice (Crossnohere et al., 2023) as well as in clinical trial design and reporting (Calvert et al., 2013); *(ii)* specific guidelines for N-of-1 clinical trials should address some of the personalised features of traditional medicine (Li et al., 2019); *(iii)* the traditional theories of *“using different treatments for the same disease and the same treatment for different diseases”* could be addressed by combining syndrome differentiation-based stratified diagnosis and Master Protocols, including Basket, Umbrella and Platform trials, which are guided by the CONSORT-ROUTINE guideline (Kwakkenbos et al., 2021); (*iv*) importantly, any modernised traditional medicines must be carefully studied in comparison with their corresponding traditional formulations to demonstrate any potential advantage in safety and efficacy, to clarify active components, and to ensure stable chemical profiles and activities through implementing Good Agricultural, Collection, and Manufacturing practices, rather than purely aim at commercial benefits; (*v*) to truly modernise, we must look beyond product regulation and include research, practitioners and practices, with the goal to harness traditional and complementary medicine for health promotion; and (*vi*) the integration of valid traditional practices into health systems would certainly be a key resource for reorienting care from a disease-focused to a person-centred model (Von Schoen-Angerer et al., 2023).

Finally, as most of the current IS/IGPG are consensus, rather than evidence-based, implementation science will be needed to examine whether they have achieved their goals in supporting the development of high-quality research evidence (Davidson et al., 2013) and to refine them in the light of new evidence.

### 4.2 International producers and depositories of IS/IGPG

We identified 33 producers and depositories, among which ICH, WHO, EQUATOR Network, WFAS and EMA were the leading producers of IGPG (Figure 2A), while EDQM, EUM, AU, EMA and ISO were leaders in publishing IS, particularly monographs of herbal drugs (Figure 2B). Although these international organisations collaborate periodically, their overall goals, tasks and statuses differ. Consequently, they publish complementary IS/IGPG, as exemplified by the WHO and the World Federation of Chinese Medicine Societies (WFCMS) jointly standardising TCM terminologies (World Health Organization, 2007d; World Federation of Acupuncture-Moxibustion Societies, 2008; World Health Organization, 2022b; Xu, 2023). The TCIM-related extension guidelines deposited at the EQUATOR Network focus on reporting transparency and quality of clinical and experimental studies (Enhancing the QUAlity and Transparency Of health Research), while WHO guidelines focus on global strategies, terminology, nomenclature, diagnosis, training benchmark and practice guidelines, including good manufacturing practices (GMP); ISO collaborated with WFCMS in publishing more than 100 standards of TCM products and has more recently started to develop Indian medicine-focused IS. Meanwhile, EMA, EDQM and ICH standards and guidelines are more of regulatory nature.

Focusing on European standards from the EMA, EDQM and ESCOP provides another excellent illustration of the division oflabour between different standards bodies and guideline providers in the field of TCIM. Through the EMA, the European Union has established guidelines and directives for traditional and well-established herbal drugs and preparations, which are legally binding for marketing authorisation in 27 European countries. EMA monographs have already covered more than 160 herbal drugs, focusing on efficacy and safety. The EDQM, an institution of the European Council, oversees the elaboration and publication of monographs for the European Pharmacopoeia (Ph. Eur.), which is legally binding in its 41 member states. It contains the quality standards of 346 herbal drugs and herbal drug preparations, plus 8 general guidelines and 41 individual monographs defining the quality of homoeopathic preparations (European Pharmacopoeia, 2022). Since 2015, the Eurasian Economic Union (EAEU) has been established to develop an integrated single market. The EAEU has passed directives to harmonise herbal quality standards (Eurasian Economic Union, 2016a; Eurasian Economic Union, 2016b; Eurasian Economic Union, 2018a; Eurasian Economic Union, 2018b; Eurasian Economic Union, 2018c; Eurasian Economic Union, 2019a; Eurasian Economic Union, 2019b; Eurasian Economic Union, 2019c; Eurasian Economic Union, 2021a; Eurasian Economic Union, 2021b; Eurasian Economic Union, 2022) and national pharmacopoeias (Eurasian Economic Union, 2020) among member states, including Armenia, Belarus, Kazakhstan, Kyrgyzstan and Russia (Whaley et al., 2023; Frolova et al., 2024; Olenina, 2025). In contrast to these regulatory bodies, the ESCOP is an umbrella organisation representing national herbal medicine or phytotherapy societies across Europe. It has so far published 85 monographs, which review the therapeutic use and scientific evidence of herbal drugs used in European phytotherapy (The European Scientific Cooperative on Phytotherapy, 2003; The European Scientific Cooperative on Phytotherapy, 2009) (Figures 2A,B). Though not legally binding, these monographs are important guidelines for clinical use and scientific research.

To sustain quality, it is essential that the most updated IS/IGPG are accessed from the websites of the relevant IS/IGPG producers (Figures 2A, 2B; Suppl. Table 4) (International Council for Harmonization of Technical Requirements for Pharmaceuticals for Human Use, 2000a; International Council for Harmonization of Technical Requirements for Pharmaceuticals for Human Use, 2000b; International Council for Harmonization of Technical Requirements for Pharmaceuticals for Human Use, 2000c; World Health Organization, 2000a; World Health Organization, 2000b; Macpherson et al., 2001; International Council for Harmonization of Technical Requirements for Pharmaceuticals for Human Use, 2002; World Health Organization, 2002b; International Council for Harmonization of Technical Requirements for Pharmaceuticals for Human Use, 2003a; International Council for Harmonization of Technical Requirements for Pharmaceuticals for Human Use, 2003b; International Council for Harmonization of Technical Requirements for Pharmaceuticals for Human Use, 2003c; World Health Organization, 2003a; World Health Organization, 2003b; World Health Organization, 2003c; World Health Organization, 2003d; International Council for Harmonization of Technical Requirements for Pharmaceuticals for Human Use, 2004a; International Council for Harmonization of Technical Requirements for Pharmaceuticals for Human Use, 2004b; World Health Organization, 2004a; World Health Organization, 2004b; European Medicines Agency, 2005; International Council for Harmonization of Technical Requirements for Pharmaceuticals for Human Use, 2005a; International Council for Harmonization of Technical Requirements for Pharmaceuticals for Human Use, 2005b; International Council for Harmonization of Technical Requirements for Pharmaceuticals for Human Use, 2005c; Liu et al., 2005; European Medicines Agency, 2006a; European Medicines Agency, 2006b; Gagnier et al., 2006; International Council for Harmonization of Technical Requirements for Pharmaceuticals for Human Use, 2006a; International Council for Harmonization of Technical Requirements for Pharmaceuticals for Human Use, 2006b; Cassileth et al., 2007; Dean et al., 2007; Deng et al., 2007; International Council for Harmonization of Technical Requirements for Pharmaceuticals for Human Use, 2007a; International Council for Harmonization of Technical Requirements for Pharmaceuticals for Human Use, 2007b; Von Elm et al., 2007; World Health Organization, 2007a; World Health Organization, 2007b; World Health Organization, 2007c; European Medicines Agency, 2008a; European Medicines Agency, 2008b; International Council for Harmonization of Technical Requirements for Pharmaceuticals for Human Use, 2008a; International Council for Harmonization of Technical Requirements for Pharmaceuticals for Human Use, 2008b; World Health Organization, 2008; Deng et al., 2009; International Council for Harmonization of Technical Requirements for Pharmaceuticals for Human Use, 2009a; International Council for Harmonization of Technical Requirements for Pharmaceuticals for Human Use, 2009b; International Council for Harmonization of Technical Requirements for Pharmaceuticals for Human Use, 2009c; Kelly et al., 2009; World Health Organization, 2009; European Medicines Agency, 2010a; European Medicines Agency, 2010b; European Medicines Agency, 2010c; International Council for Harmonization of Technical Requirements for Pharmaceuticals for Human Use, 2010a; International Council for Harmonization of Technical Requirements for Pharmaceuticals for Human Use, 2010b; International Council for Harmonization of Technical Requirements for Pharmaceuticals for Human Use, 2010c; International Council for Harmonization of Technical Requirements for Pharmaceuticals for Human Use, 2010d; International Council for Harmonization of Technical Requirements for Pharmaceuticals for Human Use, 2010e; International Council for Harmonization of Technical Requirements for Pharmaceuticals for Human Use, 2010f; International Council for Harmonization of Technical Requirements for Pharmaceuticals for Human Use, 2010g; International Council for Harmonization of Technical Requirements for Pharmaceuticals for Human Use, 2010h; International Council for Harmonization of Technical Requirements for Pharmaceuticals for Human Use, 2010i; International Council for Harmonization of Technical Requirements for Pharmaceuticals for Human Use, 2010j; International Council for Harmonization of Technical Requirements for Pharmaceuticals for Human Use, 2010k; International Council for Harmonization of Technical Requirements for Pharmaceuticals for Human Use, 2010l; International Council for Harmonization of Technical Requirements for Pharmaceuticals for Human Use, 2010m; International Council for Harmonization of Technical Requirements for Pharmaceuticals for Human Use, 2010n; Macpherson et al., 2010; Moher et al., 2010; Balshem et al., 2011; Bian and Chang, 2011; European Medicines Agency, 2011; Guyatt, G. et al., 2011; Guyatt, G. H. et al., 2011; World Health Organization, 2011a; World Health Organization, 2011b; Society for Acupuncture Research, 2011-2016; Council for International Organizations of Medical Sciences, 2012; International Council for Harmonization of Technical Requirements for Pharmaceuticals for Human Use, 2012a; International Council for Harmonization of Technical Requirements for Pharmaceuticals for Human Use, 2012b; International Council for Harmonization of Technical Requirements for Pharmaceuticals for Human Use, 2012c; International Council for Harmonization of Technical Requirements for Pharmaceuticals for Human Use, 2012d; International Federation of Orthopaedic Manipulative Physical Therapists, 2012; Qaseem et al., 2012; Welch et al., 2012; World Health Organization, 2012a; World Health Organization, 2012b; Beller et al., 2013; Chan et al., 2013; Cheng et al., 2013; International Council for Harmonization of Technical Requirements for Pharmaceuticals for Human Use, 2013; Council for International Organizations of Medical Sciences, 2014; Greenlee et al., 2014; International Organization for Standardization, 2014a; International Organization for Standardization, 2014b; International Organization for Standardization, 2014c; International Organization for Standardization, 2014d; Munk and Boulanger, 2014; Witt et al., 2014; Danan and Teschke, 2015; Hutton et al., 2015; International Organization for Standardization, 2015a; International Organization for Standardization, 2015b; International Organization for Standardization, 2015c; International Organization for Standardization, 2015d; Moher et al., 2015; Stewart et al., 2015; Council for International Organizations of Medical Sciences, 2016a; Council for International Organizations of Medical Sciences, 2016b; European Medicines Agency, 2016a; European Medicines Agency, 2016b; Heidari et al., 2016; International Council for Harmonization of Technical Requirements for Pharmaceuticals for Human Use, 2016; International Federation of Orthopaedic Manipulative Physical Therapists, 2016a; International Federation of Orthopaedic Manipulative Physical Therapists, 2016b; International Organization for Standardization, 2016a; International Organization for Standardization, 2016b; International Organization for Standardization, 2016c; International Organization for Standardization, 2016d; Lachat et al., 2016; Ogrinc et al., 2016; World Health Organization, 2016a; World Health Organization, 2016b; Zorzela et al., 2016; Biganzoli et al., 2017; Chen, Y. et al., 2017a; Chen, Yaolong et al., 2017b; Cheng et al., 2017; European Medicines Agency, 2017; Greenlee et al., 2017; Guise et al., 2017; International Council for Harmonization of Technical Requirements for Pharmaceuticals for Human Use, 2017a; International Council for Harmonization of Technical Requirements for Pharmaceuticals for Human Use, 2017b; International Council for Harmonization of Technical Requirements for Pharmaceuticals for Human Use, 2017c; International Federation of Orthopaedic Manipulative Physical Therapists, 2017; International Organization for Standardization, 2017a; International Organization for Standardization, 2017b; International Organization for Standardization, 2017c; International Organization for Standardization, 2017d; International Organization for Standardization, 2017e; International Organization for Standardization, 2017f; International Organization for Standardization, 2017g; International Organization for Standardization, 2017h; International Organization for Standardization, 2017i; International Organization for Standardization, 2017j; International Organization for Standardization, 2017k; International Organization for Standardization, 2017l; International Organization for Standardization, 2017m; International Organization for Standardization, 2017n; International Organization for Standardization, 2017o; International Organization for Standardization, 2017p; International Organization for Standardization, 2017q; Chen et al., 2017a; World Health Organization, 2017; World Health Organization, 2023; Committee on Publication Ethics Guidelines, 2018; Heinrich et al., 2018; International Organization for Standardization, 2018a; International Organization for Standardization, 2018b; International Organization for Standardization, 2018c; International Organization for Standardization, 2018d; International Organization for Standardization, 2018e; International Organization for Standardization, 2018f; International Organization for Standardization, 2018g; International Organization for Standardization, 2018h; International Organization for Standardization, 2018i; World Health Organization, 2018a; World Health Organization, 2018b; Dai et al., 2019; European Medicines Agency, 2019; International Council for Harmonization of Technical Requirements for Pharmaceuticals for Human Use, 2019a; International Council for Harmonization of Technical Requirements for Pharmaceuticals for Human Use, 2019b; International Council for Harmonization of Technical Requirements for Pharmaceuticals for Human Use, 2019c; International Organization for Standardization, 2019a; International Organization for Standardization, 2019b; International Organization for Standardization, 2019c; International Organization for Standardization, 2019d; International Organization for Standardization, 2019e; International Organization for Standardization, 2019f; International Organization for Standardization, 2019g; International Organization for Standardization, 2019h; International Organization for Standardization, 2019i; International Organization for Standardization, 2019j; International Organization for Standardization, 2019k; International Organization for Standardization, 2019l; International Organization for Standardization, 2019m; International Organization for Standardization, 2019n; International Organization for Standardization, 2019o; International Organization for Standardization, 2019p; International Organization for Standardization, 2019q; International Organization for Standardization, 2019r; Tang et al., 2019; Wang et al., 2019; World Health Organization, 2019a; World Health Organization, 2019b; International Council for Harmonization of Technical Requirements for Pharmaceuticals for Human Use, 2020; International Organization for Standardization, 2020a; International Organization for Standardization, 2020b; International Organization for Standardization, 2020c; International Organization for Standardization, 2020d; International Organization for Standardization, 2020e; International Organization for Standardization, 2020f; International Organization for Standardization, 2020g; International Organization for Standardization, 2020h; International Organization for Standardization, 2020i; International Organization for Standardization, 2020j; International Organization for Standardization, 2020k; International Organization for Standardization, 2020l; International Organization for Standardization, 2020m; International Organization for Standardization, 2020n; International Organization for Standardization, 2020o; International Organization for Standardization, 2020p; International Organization for Standardization, 2020q; International Organization for Standardization, 2020r; International Organization for Standardization, 2020s; International Organization for Standardization, 2020t; International Organization for Standardization, 2020u; Mcgowan et al., 2020; Percie Du Sert et al., 2020; Society for Integrative Oncology, 2020; World Health Organization, 2020a; World Health Organization, 2020b; World Health Organization, 2020c; Xie et al., 2020; Zhang et al., 2020a; Zhang et al., 2020b; Zhang et al., 2020c; International Council for Harmonization of Technical Requirements for Pharmaceuticals for Human Use, 2021; International Organization for Standardization, 2021a; International Organization for Standardization, 2021b; International Organization for Standardization, 2021c; International Organization for Standardization, 2021d; International Organization for Standardization, 2021e; International Organization for Standardization, 2021f; International Organization for Standardization, 2021g; International Organization for Standardization, 2021h; International Organization for Standardization, 2021i; International Organization for Standardization, 2021j; International Organization for Standardization, 2021k; International Organization for Standardization, 2021l; International Organization for Standardization, 2021m; Page et al., 2021; Rethlefsen et al., 2021; Tang et al., 2021; Council for International Organizations of Medical Sciences, 2022; European Medicines Agency, 2022a; European Medicines Agency, 2022b; European Medicines Agency, 2022c; Husereau et al., 2022; International Council for Harmonization of Technical Requirements for Pharmaceuticals for Human Use, 2022a; International Council for Harmonization of Technical Requirements for Pharmaceuticals for Human Use, 2022b; International Council for Harmonization of Technical Requirements for Pharmaceuticals for Human Use, 2022c; International Council for Harmonization of Technical Requirements for Pharmaceuticals for Human Use, 2022d; International Council for Harmonization of Technical Requirements for Pharmaceuticals for Human Use, 2022e; International Organization for Standardization, 2022a; International Organization for Standardization, 2022b; International Organization for Standardization, 2022c; International Organization for Standardization, 2022d; International Organization for Standardization, 2022e; International Organization for Standardization, 2022f; International Organization for Standardization, 2022g; International Organization for Standardization, 2022h; International Organization for Standardization, 2022i; International Organization for Standardization, 2022j; International Organization for Standardization, 2022k; International Organization for Standardization, 2022l; International Organization for Standardization, 2022m; International Organization for Standardization, 2022n; International Organization for Standardization, 2022o; International Organization for Standardization, 2022p; International Organization for Standardization, 2022q; International Organization for Standardization, 2022r; International Organization for Standardization, 2022s; International Organization for Standardization, 2022t; International Organization for Standardization, 2022u; Mao et al., 2022a; Mao et al., 2022b; Song et al., 2022; Ward et al., 2022; World Health Organization, 2022a; Association for Applied Psychophysiology and Biofeedback, 2023; Carlson et al., 2023; Christensen et al., 2023; Crossnohere et al., 2023; Digital Therapeutics Alliance, 2023; International Council for Harmonization of Technical Requirements for Pharmaceuticals for Human Use, 2023a; International Council for Harmonization of Technical Requirements for Pharmaceuticals for Human Use, 2023b; International Council for Harmonization of Technical Requirements for Pharmaceuticals for Human Use, 2023c; International Organization for Standardization, 2023a; International Organization for Standardization, 2023b; International Organization for Standardization, 2023c; International Organization for Standardization, 2023d; International Organization for Standardization, 2023e; International Organization for Standardization, 2023f; International Organization for Standardization, 2023g; International Organization for Standardization, 2023h; International Organization for Standardization, 2023i; International Organization for Standardization, 2023j; International Organization for Standardization, 2023k; Ma et al., 2023; The Wildlife Trade Monitoring Network, 2023; World Federation of Acupuncture-Moxibustion Societies, 2023a; World Federation of Acupuncture-Moxibustion Societies, 2023b; World Federation of Acupuncture-Moxibustion Societies, 2023c; World Federation of Acupuncture-Moxibustion Societies, 2023d; World Federation of Acupuncture-Moxibustion Societies, 2023e; World Federation of Acupuncture-Moxibustion Societies, 2023f; World Federation of Acupuncture-Moxibustion Societies, 2023g; World Federation of Acupuncture-Moxibustion Societies, 2023h; World Federation of Acupuncture-Moxibustion Societies, 2023i; World Federation of Acupuncture-Moxibustion Societies, 2023j; World Federation of Acupuncture-Moxibustion Societies, 2023k; World Federation of Acupuncture-Moxibustion Societies, 2023l; World Federation of Acupuncture-Moxibustion Societies, 2023m; World Federation of Acupuncture-Moxibustion Societies, 2023n; World Federation of Acupuncture-Moxibustion Societies, 2023o; World Federation of Acupuncture-Moxibustion Societies, 2023p; World Federation of Acupuncture-Moxibustion Societies, 2023q; World Federation of Acupuncture-Moxibustion Societies, 2023r; World Federation of Acupuncture-Moxibustion Societies, 2023s; World Health Organization, 2023a; Zhang et al., 2023; Akl et al., 2024; Association of Official Agricultural Chemists International, 2024; Bower et al., 2024; Carlson et al., 2024; Healthcare Information and Management Systems Society, 2024; International Council for Harmonization of Technical Requirements for Pharmaceuticals for Human Use, 2024a; International Council for Harmonization of Technical Requirements for Pharmaceuticals for Human Use, 2024b; International Council for Harmonization of Technical Requirements for Pharmaceuticals for Human Use, 2024c; International Council for Harmonization of Technical Requirements for Pharmaceuticals for Human Use, 2024d; International Organization for Standardization, 2024a; International Organization for Standardization, 2024b; International Organization for Standardization, 2024c; International Organization for Standardization, 2024d; International Organization for Standardization, 2024e; International Organization for Standardization, 2024f; International Organization for Standardization, 2024g; International Organization for Standardization, 2024h; International Organization for Standardization, 2024i; International Organization for Standardization, 2024j; International Organization for Standardization, 2024k; International Organization for Standardization, 2024l; International Organization for Standardization, 2024m; International Organization for Standardization, 2024n; International Organization for Standardization, 2024o; International Organization for Standardization, 2024p; International Organization for Standardization, 2024q; Li et al., 2024; Rubio et al., 2024; World Federation of Acupuncture-Moxibustion Societies, 2024; World Health Organization, 2024a; World Health Organization, 2024b; World Health Organization, 2024c; International Committee of Medical Journal Editors, 2025; International Organization for Standardization, 2025a; International Organization for Standardization, 2025b; International Organization for Standardization, 2025c; International Organization for Standardization, 2025d; Schünemann et al., 2025; Sousa-Pinto et al., 2025; Guidelines International Network, NA; International Organization for Standardization, NA; Society for Acupuncture Research, NA; World Health Organization, 2026a; World Health Organization, 2026b; World Health Organization, 2026c; World Health Organization, 2026d; World Health Organization, 2026e; World Health Organization, 2026f) (European Pharmacopoeia, 2022; The Gulf Health Council, 2022; European Medicines Agency, 2004).

To date, IS/IGPG have only been developed for a limited number of TCIM modalities (Figure 2C). Future efforts are expected to expand TCIM coverage to a wider range of therapies, whenever feasible. While wider stakeholder participation is crucial for such extensions, the WHO emphasises the need for safeguards that respect indigenous rights and protect traditional knowledge from misappropriation, which remains an unresolved challenge (Burki, 2025). Looking forward, WHO will need to enhance coordination among its member states to encourage consistency in allocating available resources for research on TCIM; indeed, many countries still fail to adequately invest in TCIM research, which prevents the generation of evidence relevant at the local level and limits integration of traditional medicines into national health systems (Von Schoen-Angerer et al., 2023). Additionally, applying mainstream clinical research standards, which are often cost-prohibitive and structurally misaligned with TCIM paradigms, may not be appropriate for authentic TCIM practice. Indeed, effective models for integrating traditional medicine into healthcare systems are often built upon a foundation of established traditional use, even in the absence of robust RCTs. This is evident in the recognition of Ayurveda in India, TCM in China, and the registration scheme for traditional herbal medicines in Europe. These nuances of traditional medicine integration are worthy of consideration, indicating the need for specialised IS/IGPG tailored to TCIM to respect their conceptual diversity, traditional use histories, and holistic approaches, while guaranteeing quality, safety and cost-effectiveness.

### 4.3 IS/IGPG users and usage

Despite the complexity of TCIM, its combination with new technologies, such as omics (Chen et al., 2024) or AI (Wang et al., 2025; World Health Organization, 2025b), brings about many promising opportunities. Beyond recurring issues with botanical and pharmacopoeial nomenclature and quality (Rivera et al., 2014; Heinrich et al., 2022; Wang et al., 2023), a lack of adherence to existing IS/IGPG commonly results in low-quality and biased research data (Supplementary Table S5). The accessibility to existing IS/IGPG can be a major obstacle. Many documents exist in the form of unindexed grey literature, requiring considerable effort, multilingual skills, and information literacy to retrieve. Cost disparities also hinder access: while WHO and some organisations provide free resources, ISO and other bodies charge fees, creating difficulties for low-income regions, organisations and individuals that cannot afford the costs of up-to-date standards.

The value of IS/IGPG can only be fully appreciated by considering their implementation at multiple levels: in basic sciences, medical research, and therapy. It requires adaptation of science and health policies and their effective auditing, as well as effective use by researchers and practitioners. Therefore, a global disparity that appears in the use of these tools, dominated by leading economies, is a cause for concern (Supplementary Table S5). Given the importance of TCIM in the developing world, this lack of use of existing IS/IGPG in this major part of the world calls for urgent attention. Overcoming language barriers may also be crucial. Of note, the EQUATOR Network makes a step in that direction; however, as of 5 October 2025, only a small proportion of its guidelines (688) have been translated into 17 languages. For example, only 25 guidelines have official translations in Chinese (Equator Network, NA). More official translations and dissemination activities should increase awareness, endorsement and implementation.

There are many approaches to promoting the dissemination and implementation of IS/IGPG in the field of TCIM, for which Hong Kong’s methodologies for policy guidance may offer valuable inspiration. These include *(i)* applying the CIFR framework to identify barriers and facilitators for improving guidance uptake (Damschroder et al., 2009); *(ii)* utilising the RE-AIM framework to enhance dissemination, outreach and implementation (Glasgow et al., 1999; Lam et al., 2022); and *(iii)* developing focus groups and Delphi surveys to inform policies (Lam et al., 2022).

At the 78th World Health Assembly on 27 May 2025, the WHO has made it a priority to strengthen national capacities in evidence-based decision-making for the adoption and effective application of norms and standards. It can then be expected that the WHO is to play a leading role in a proactive global dissemination of IS/IGPG. Governmental agencies, charitable funding bodies, healthcare associations, international societies, like the GP-TCM Research Association (https://www.gp-tcm.org/), and individual scholars all have a role to play.

### 4.4 Limitations

This search using English keywords and MeSH terms was limited to publications between 2000 and 2025, which precludes the coverage of more classic IS and IGPG, *e.g*., the WHO guidelines on quality, safety, efficacy, research, rational use and conservation of medicinal plants published before 2000 (World Health Organization, 1991). Searches were also limited by the availability of grey literature on organisational websites, meaning omissions may have occurred despite combining this with systematic searches of five databases, AI-assisted systematic retrieval and human expert input. Through expert input, IS/IGPG publications in non-English languages were included, *e.g.*, *(i)* more than 20 WFCMS guidelines on TCM prescription, dispensing, delivery, decoction and administration, which are published in Chinese with or without an English title (World Federation of Chinese Medicine Societies, 2022-2026); and *(ii)* the Spanish-language Mexican herbal pharmacopoeia, that is binding for several Latin American countries (Estados Unidos Mexicanos, 2021a; Estados Unidos Mexicanos, 2021b). Nonetheless, our team lacked experts from the Americas, Southern parts of Africa, as well as Middle Eastern, Oceanic, and ASEAN countries, which may have limited the intended worldwide coverage, especially as it relates to non-English documents and grey literature. Although this scoping review focuses on international standards and guidelines, it does not imply that national or regional guidelines are not important. In fact, many regulatory standards and practice guidelines are not yet harmonised internationally. In such contexts, national and regional standards and guidelines should be considered, observed and fully respected. Furthermore, we cannot exclude the possibility that the Category (b) dataset across the 14,999 studies listed in Supplementary Table S5 may involve overlapping data, as previously reported by Rizzo *et al* (Rizzo et al., 2025). Finally, as with any scoping review, a major limitation is the lack of formal assessment of the quality and impact of included documents. Both of these factors are crucial and should be addressed in future studies.

## 5 Conclusion

This scoping review has mapped the development of IS/IGPG within the TCIM field and created a comprehensive, accessible repository, thereby fulfilling its primary objectives. It has also compiled a database of secondary documents at the intersection of TCIM and IS/IGPG to inform future work, which includes raising awareness, promoting endorsement, implementation and harmonisation of IS/IGPG, as well as evaluating the effectiveness of these standards and guidelines in supporting evidence-based TCIM worldwide.

## Glossary

AAPB: Association for Applied Psychophysiology and Biofeedback
AI: Artificial Intelligence
AOAC: Association of Official Agricultural Chemists International
AU: African Union
CA: Comunidad Andina (*La Comunidad Andina*)
CIOMS: Council for International Organizations of Medical Sciences
COPE: Committee on Publication Ethics guidelines
DTx: Digital Therapeutics Alliance
EAEU: Eurasian Economic Union
EDQM: European Directorate for the Quality of Medicines and HealthCare
EMA/HMPC: European Medicines Agency/Committee on Herbal Medicinal Products
EQUATOR Network: Enhancing the QUAlity and Transparency Of health Research
ESCOP: European Scientific Cooperative on Phytotherapy
EUM: Estados Unidos Mexicanos
EUSOMA: European Society of Breast Cancer Specialists
GA: Society for Medicinal Plant and Natural Product Research
GBIF: Global Biodiversity Information Facility
GCRSR: Global Coalition for Regulatory Science Research
GHC/GCC: The Gulf Health Council/The Gulf Cooperation Council
GIN: Guidelines International Network
GMP: Good Manufacturing Practices
HIMSS: Healthcare Information and Management Systems Society
HL7 International: Health Level Seven International
HPLC: High-Performance Liquid Chromatography
HRC: The Pacific Health Research Committee and the Health Research Council of New Zealand
ICC: Information, Concepts and Context
ICD-11: International Classification of Diseases, 11^th^ Revision
ICH: International Council for Harmonization of Technical Requirements for Pharmaceuticals for Human Use
ICMJE: International Federation of Orthopaedic Manipulative Physical Therapist
IGPG: International Good Practice Guidelines
IS: International Standards
ISCMR: International Society for Complementary Medicine Research
ISE: International Society for Ethnopharmacology
ISO: International Organisation for Standardisation
LAP: Latin American Parliament
LLM: Large Language Model
MMPT: Manual and Musculoskeletal Physical Therapies
NS: Not Specific, But Nonetheless Highly Relevant to TCIM
OMPT: Orthopaedic Manipulative Physical Therapies
PAHO: Pan American Health Organization
PCC: Population, Concept and Context
Ph. Eur.: European Pharmacopoeia
SAR: Society for Acupuncture Research
SIO: Society for Integrative Oncology
SIOP: International Society of Paediatric Oncology
TCIM: Traditional, Complementary and Integrative Medicine
TCIMg: General IS/IGPG in the Broad Field of Traditional, Complementary and Integrative Medicine
TCM: Traditional Chinese Medicine
TCMg: General IS/IGPG in the Field of Traditional Chinese Medicine
TRAFFIC: The long-term Vision of the Kunming-Montreal Global Biodiversity Framework
WAHO: West African Health Organization
WFAS: World Federation of Acupuncture-Moxibustion Societies
WFCMS: World Federation of Chinese Medicine Societies
WHO: World Health Organization
