# Supplementary material for: International standards and good practice guidelines in traditional, complementary and integrative medicine: a scoping review
Source: Front Pharmacol. 2026 Apr 9;17:1742400. doi: 10.3389/fphar.2026.1742400 (PMC13102575; doi:10.3389/fphar.2026.1742400)
Supplement: Supplementary file 1 [file Supplementaryfile1.zip › Supplementary Tables 1, 2, 3, and 6.docx]

Supplementary Material

1. **Suppl. TABLE S1.** Strategies for data retrieval.

| Database | Search strategy |
| --- | --- |
| PubMed, and Web of Science | (((("complementary therapies"[Mesh] OR "medicine, traditional"[Mesh] OR "integrative medicine"[Mesh]) OR (("alternative medicine"[tiab] OR "complementary and alternative medicine"[tiab] OR "CAM"[tiab] OR "integrated medicine"[tiab]) OR ("traditional medicine"[tiab] OR "herbal medicine"[tiab] OR "phytotherapy"[tiab]))) AND ("guidelines as topic"[Mesh] OR "practice guidelines as topic"[Mesh] OR "standards"[Subheading] OR ("guidelines"[tiab] OR "standards"[tiab] OR "good practice*"[tiab] OR "quality control"[tiab] OR "research ethics"[tiab]))) OR (("biomedical research"[Mesh:NoExp] OR "research design"[Mesh] OR ("polymerase chain reaction"[Mesh] OR "high-throughput nucleotide sequencing"[Mesh] OR "clinical trials as topic"[Mesh] OR "observational studies as topic"[Mesh] OR "systematic reviews as topic"[Mesh] OR "review literature as topic"[Mesh])) AND ("guidelines as topic"[Mesh] OR "practice guidelines as topic"[Mesh] OR ("guidelines"[tiab] OR "standards"[tiab] OR "good practice*"[tiab])) AND (("traditional medicine"[tiab] OR "complementary medicine"[tiab] OR "integrative medicine"[tiab]) OR "integrative medicine"[Mesh]))) NOT ("animals"[Mesh] NOT "humans"[Mesh])  Filters: 20000101 to 20250430 (YYYY-MM-DD) |
| ProQuest | "(((("complementary therapies"[Mesh] OR "medicine, traditional"[Mesh] OR "integrative medicine"[Mesh]) OR (("alternative medicine"[tiab] OR "complementary and alternative medicine"[tiab] OR "CAM"[tiab] OR "integrated medicine"[tiab]) OR ("traditional medicine"[tiab] OR "herbal medicine"[tiab] OR "phytotherapy"[tiab]))) AND ("guidelines as topic"[Mesh] OR "practice guidelines as topic"[Mesh] OR "standards"[Subheading] OR ("guidelines"[tiab] OR "standards"[tiab] OR ("good practice" OR "good practices")[tiab] OR "quality control"[tiab] OR "research ethics"[tiab]))) OR (("biomedical research"[Mesh:NoExp] OR "research design"[Mesh] OR ("polymerase chain reaction"[Mesh] OR "high-throughput nucleotide sequencing"[Mesh] OR "clinical trials as topic"[Mesh] OR "observational studies as topic"[Mesh] OR "systematic reviews as topic"[Mesh] OR "review literature as topic"[Mesh])) AND ("guidelines as topic"[Mesh] OR "practice guidelines as topic"[Mesh] OR ("guidelines"[tiab] OR "standards"[tiab] OR " good practice*"[tiab])) AND (("traditional medicine"[tiab] OR "complementary medicine"[tiab] OR "integrative medicine"[tiab]) OR "integrative medicine"[Mesh]))) NOT ("animals"[Mesh] NOT "humans"[Mesh])"  Filters: 20000101 to 20250430 (YYYY-MM-DD) |
| EMBASE | ('traditional medicine'/exp OR 'traditional medicine' OR 'alternative medicine'/exp OR 'alternative medicine' OR 'integrative medicine'/exp OR 'integrative medicine') AND ('practice guideline'/exp OR 'practice guideline' OR 'standard'/exp OR 'standard') NOT ('animal'/exp OR 'animal') AND [2000-2025]/py  Filters: 20000101 to 20250430 (YYYY-MM-DD) |
| Cochrane Library | (((("complementary therapies"[Mesh] OR "medicine, traditional"[Mesh] OR "integrative medicine"[Mesh]) OR (("alternative medicine"[tiab] OR "complementary and alternative medicine"[tiab] OR "CAM"[tiab] OR "integrated medicine"[tiab]) OR ("traditional medicine"[tiab] OR "herbal medicine"[tiab] OR "phytotherapy"[tiab]))) AND ("guidelines as topic"[Mesh] OR "practice guidelines as topic"[Mesh] OR "standards"[Subheading] OR ("guidelines"[tiab] OR "standards"[tiab] OR "good practice*"[tiab] OR "quality control"[tiab] OR "research ethics"[tiab]))) OR (("biomedical research"[Mesh:NoExp] OR "research design"[Mesh] OR ("polymerase chain reaction"[Mesh] OR "high-throughput nucleotide sequencing"[Mesh] OR "clinical trials as topic"[Mesh] OR "observational studies as topic"[Mesh] OR "systematic reviews as topic"[Mesh] OR "review literature as topic"[Mesh])) AND ("guidelines as topic"[Mesh] OR "practice guidelines as topic"[Mesh] OR ("guidelines"[tiab] OR "standards"[tiab] OR "good practice*"[tiab])) AND (("traditional medicine"[tiab] OR "complementary medicine"[tiab] OR "integrative medicine"[tiab]) OR "integrative medicine"[Mesh]))) NOT ("animals"[Mesh] NOT "humans"[Mesh]).  Filters: 20000101 to 20250430 (YYYY-MM-DD) |
| Grey literature | Data extraction design: using a large language model (LLM), Claude Opus 4 (Anthropic, San Francisco, CA, USA).   1. **Scoping and source enumeration**   To ensure that the survey of standards was truly comprehensive, we established six primary “seed” entry points, each chosen for its authority and for the distinctive segment of the regulatory landscape it represents.  First, we used the “International Standards & Guidelines” index curated by King’s Centre for Integrative Chinese Medicine. This page offers a continuously updated, practitioner-focused compilation of links that already spans WHO, ISO/TC 249, WFCMS and a range of research-reporting checklists relevant to Traditional, Complementary and Integrative Medicine (TCIM).  Second, we accessed the World Health Organization’s Traditional Medicine programme portal, the official repository for WHO Benchmarks (training and practice), GACP and GMP guidelines, the ICD-11 TM chapter and global policy strategy documents.  Third, we drew directly on the ISO/TC 249 public workspace, which maintains the definitive catalogue of ISO standards covering herbal raw-material quality, heavy-metal testing, decoction-service requirements and clinical-practice specifications.  Fourth, we harvested the World Federation of Chinese Medicine Societies (WFCMS) standards list. The WFCMS SCM series supplies internationally recognised operational standards—including curriculum frameworks and technique-specific operating procedures—that are not mirrored on ISO or WHO sites.  Fifth, to capture research-methodology guidance, we incorporated two specialised hubs: the EQUATOR Network checklist library (home to CONSORT extensions, STRICTA, PRISMA-CHM and related tools) and the NC3Rs resource centre, which houses the ARRIVE and PREPARE guidelines for animal studies.  Finally, we included biomedical-ethics and publication-integrity statements hosted by the World Medical Association (e.g., the Declarations of Helsinki and Taipei) and by the Committee on Publication Ethics (COPE), ensuring that fundamental human-subject and editorial principles were represented alongside technical standards.  From each seed page the crawler followed first- and second-order hyperlinks. A document was retained only when two conditions were simultaneously met: (i) the landing URL resided on an official or publisher-verified domain—such as .who.int, .iso.org, .wfcms.org, .ema.europa.eu, .equatornetwork.org, .nc3rs.org.uk, .wma.net or .publicationethics.org—and (ii) the file itself was a standard, guideline or formal reporting checklist that demonstrably governs TCIM substances, practices, research methods or publication ethics. This multi-seed strategy expanded the retrieval universe well beyond the King’s index and guaranteed inclusion of key documents that would otherwise have been missed, such as the World Medical Association’s ethical declarations, NC3Rs’ ARRIVE 2.0 checklist and ISO/TC 249 draft releases.  **2. Automated retrieval** Harvesting was performed in Python 3.10 under Ubuntu 22.04. A lightweight crawler built with requests, beautifulsoup4and urllib3 recursively captured first- and second-order links; a headless Selenium/Chrome driver handled PDF files generated behind JavaScript. For each endpoint a secondary script extracted the issuing organisation, title, publication year, document type, persistent identifier and canonical HTTPS link. HTML titles were parsed via OpenGraph tags and PDF titles via their XMP /Title fields. All dates were converted to ISO 8601 format.  **3. Deduplication and cluster verification** String-level duplicates were removed with fuzzy matching (token-set ratio ≥ 95 %); residual near-duplicates were detected by embedding document titles with sentence-transformers/all-mpnet-base-v2 and clustering them with HDBSCAN. These two passes reduced 147 harvested links to 61 unique candidates, which were exported to a provisional master spreadsheet.  **4. Manual triage and authenticity audit** Two domain experts independently screened each candidate against a pre-specified grid contained in the Inclusion and Exclusion Section. The grid defined five inclusion criteria—TCIM relevance, supra-national issuing body, correct document genre, English language, and publication date between 1 January 2000 and 30 April 2025—and five parallel exclusion rules (e.g. single-nation policy only, database record, non-English language). Disagreements were resolved by a senior reviewer; Cohen’s κ was 0.87, indicating high inter-rater reliability. This step eliminated 16 grey-literature items and yielded the final corpus of 45 fully authenticated documents.  **5. Data structuring and table generation** A pandas transformer populated nine manuscript columns: Organisation, Year, Standard/Guideline, Scope, Key Parameters, Testing Methods, Typical Implementation, and URL. The resulting dataframe was saved as TCIM_Standards_Table.xlsx for supplementary material and also rendered as plain-text tables within the manuscript, guaranteeing perfect synchrony. |

1. **Suppl. TABLE S2.** Websites of the international organisations highlighted by this scoping review

| **International**   **organizations and industrial alliances** | **Full name** | **Homepage** |
| --- | --- | --- |
| AAPB | Association for Applied Psychophysiology and Biofeedback | <https://aapb.org/> |
| AOAC | Association of Official Agricultural Chemists International | <https://www.aoac.org/> |
| AU | African Union | <https://au.int/> |
| CA | Comunidad Andina (La Comunidad Andina) | https://www.comunidadandina.org/ |
| CIOMS | Council for International Organizations of Medical Sciences | <https://cioms.ch/> |
| COPE | Committee on Publication Ethics guidelines | <https://publicationethics.org/> |
| DTx | Digital Therapeutics Alliance | <https://dtxalliance.org> |
| EAEU | Eurasian Economic Union | [Eurasian Economic Union](https://eaeunion.org/?lang=en) |
| EDQM | European Directorate for the Quality of Medicines & HealthCare | https://www.edqm.eu/en/ |
| EMA/HMPC | European Medicines Agency/Committee on Herbal Medicinal Products | <https://www.ema.europa.eu/en/committees/committee-herbal-medicinal-products-hmpc> |
| EQUATOR Network | Enhancing the QUAlity and Transparency Of health Research | [https://www.equator-network.org](https://www.equator-network.org/) |
| ESCOP | European Scientific Cooperative on Phytotherapy | https://www.escop.com/ |
| EUM | Estados Unidos Mexicanos | https://farmacopea.org.mx/ |
| EUSOMA | European Society of Breast Cancer Specialists | <https://www.eusoma.org/> |
| GA | Society for Medicinal Plant and Natural Product Research | [https://ga-online.org](https://ga-online.org/) |
| GBIF | Global Biodiversity Information Facility | <https://www.gbif.org/> |
| GHC/GCC | The Gulf Health Council/ The Gulf Cooperation Council | https://www.ghc.sa/en/ |
| GCRSR | Global Coalition for Regulatory Science Research | <https://gcrsr.net/> |
| GIN | Guidelines International Network | <https://g-i-n.net/> |
| HIMSS | [Healthcare Information and Management Systems Society](https://www.himss.org/) | https://www.himss.org/ |
| HL7 International | Health Level Seven International | <https://www.hl7.org/> |
| HRC | The Pacific Health Research Committee and the Health Research Council of New Zealand | https://www.hrc.govt.nz/ |
| ICH | International Council for Harmonization of Technical Requirements for Pharmaceuticals for Human Use | <https://www.ich.org/> |
| ICMJE | International Committee of Medical Journal Editors | <https://icmje.acponline.org/> |
| IFOMPT | International Federation of Orthopaedic Manipulative Physical Therapists | <https://www.ifompt.org/> |
| ISCMR | International Society for Complementary Medicine Research | <https://www.iscmr.org/> |
| ISE | International Society for Ethnopharmacology | [https://ethnopharmacology.org](https://ethnopharmacology.org/) |
| ISO | International Organization for Standardization | [https://www.iso.org](https://www.iso.org/) |
| PAHO | Pan American Health Organization | https://iris.paho.org/discover |
| LAP | Latin American Parliament | https://parlatino.org/ |
| PROTEUS | Patient-Reported Outcomes Tools: Engaging Users and Stakeholders | https://theproteusconsortium.org/ |
| RIGHT | International Standard for Reporting Items for practice Guideline in HealThcare | <https://www.right-statement.org/> |
| SAR | Society for Acupuncture Research | <https://www.acupunctureresearch.org/> |
| SIO | Society for Integrative Oncology | <https://integrativeonc.org/> |
| SIOP | International Society of Pediatric Oncology | <https://siop-online.org/> |
| TRAFFIC | The Wildlife Trade Monitoring Network | <https://www.traffic.org/> |
| WAHO | West African Health Organization | https://www.wahooas.org/ |
| WFAS | World Federation of Acupuncture-moxibustion Societies | <https://wfas.kejie.org.cn/site-enwfas?language=en> |
| WFCMS | World Federation of Chinese Medicine Societies | <https://www.wfcms.org/en/> |
| WHO | World Health Organization | <https://www.who.int/> |

1. **Suppl. TABLE S3.** Traditional and complementary medicine subtypes and modalities

| Traditional medicine and complementary medicine |
| --- |
| acupressure |
| acupoint injection |
| acupuncture (transcutaneous electrical nerve stimulation, electrical stimulation of acupoint pc6, electroacupuncture, and pharmacopuncture) |
| Arabic (Islamic) medicine |
| aromatherapy |
| audio‐analgesia |
| auricular therapy |
| ayurveda |
| biofeedback |
| breast engorgement during lactation |
| chiropractic treatment |
| Chunsoo Korean Qi therapy |
| cognitive-behavioral treatments |
| cupping |
| Cyriax friction massage |
| electromechanical |
| energy conservation |
| exercise |
| guasha |
| healing touch |
| herbal medicines (including animals and their parts, minerals, phytotherapy, and vitamin supplementation, and dietary supplements) |
| homeopathy |
| hot compress |
| hypnosis |
| Kampo |
| Korean medicine/oriental medicine |
| low FODMAPs diet or kefir diet |
| macronutrients |
| magnesium |
| maternal massage |
| meditation |
| meridian treatment |
| mindfulness |
| motor control exercise |
| moxibustion |
| music therapy |
| natural health products |
| naturopathy |
| Oketani (breast massage in Japan) |
| physical activity |
| probiotics |
| prostatic massage |
| psychological interventions |
| qigong |
| Reiki (Usui) |
| relaxation |
| relaxation with imagery |
| scraping therapy |
| Siddha |
| Sowa-rigpa |
| specific whole foods |
| spinal manipulation |
| taiji (Tai Chi) |
| therapeutic touch |
| traditional Chinese medicine |
| traditional Indian medicine |
| tuina |
| Unani |
| wushu (martial arts) |
| yoga |

1. **Suppl. TABLE S4.** Development of IS and IGPG, from 2000 to April 2025 (**Suppl. TABLE S4** is in xlsx format)
2. **Suppl. TABLE S5.** Systematic reviews, perspectives and commentaries at the interface between IS/IGPG and TCIM (**Suppl. TABLE S5** is in xlsx format)
3. **Suppl. TABLE S6.** A comparison of the disease spectrum treated by TCIM in different eras following the WHO ICD-11 guideline (World Healt Organization, 2024)

| **Before 2002 (**World Healt Organization, 2002**)** | **2000-2025** |
| --- | --- |
| **Certain infectious or parasitic diseases**   1. Smallpox 2. Poliomyelitis 3. Malaria 4. HIV/AIDS 5. Infections | **Certain infectious or parasitic diseases**   1. Clostridium difficile-associated diarrhea (CDAD) 2. Chronic hepatitis B (HBV) 3. Chronic hepatitis C (HCV) 4. HIV/AIDS 5. COVID-19 6. H1N1 Influenza 7. Severe acute respiratory syndrome (SARS) 8. Experimental Sepsis |
| **Neoplasms**   1. Cancer | **Neoplasms**   1. Advanced or late gastric cancer 2. Breast cancer 3. Colorectal cancer 4. Oesophageal cancer 5. Cancer care 6. Cancer psychosomatic symptoms |
| **Endocrine, nutritional or metabolic diseases**   1. Diabetes 2. Exogenous obesity | **Endocrine, nutritional or metabolic diseases**   1. Adolescent obesity 2. Hyperthyroidism 3. Prediabetes 4. Type 2 diabetes (T2DM) 5. Short stature in children |
| **Mental, behavioural or neurodevelopmental disorders**   1. Anxiety 2. Panic disorders | **Mental, behavioural or neurodevelopmental disorders**   1. Anxiety 2. Depression 3. Perimenopausal depression 4. Psychological distress 5. Schizophrenia 6. Tic disorders 7. Dementia 8. Vascular dementia |
| **Sleep-wake disorders**   1. Insomnia | **Sleep-wake disorders**   1. Insomnia |
| **Diseases of the nervous system**   1. Convulsion 2. Tension migraine headache | **Diseases of the nervous system**   1. Carpal tunnel syndrome 2. Diabetic peripheral neuropathy 3. Epilepsy 4. Multiple sclerosis 5. Neuropathic pain 6. Optic atrophy 7. Parkinson's disease 8. Post-stroke 9. Poststroke cognitive impairment 10. Spasticity after stroke 11. Stroke (Cerebrovascular accident) 12. Migraine 13. Episodic migraine 14. Tension-type headache |
| **Diseases of the visual system**  NA | **Diseases of the visual system**   1. Glaucoma 2. Macular degeneration |
| **Diseases of the circulatory system**   1. Heart disease 2. Vascular diseases | **Diseases of the circulatory system**   1. Angina pectoris 2. Heart failure 3. Hypertension 4. Primary hypertension |
| **Diseases of the respiratory system**   1. Asthma | **Diseases of the respiratory system**   1. Acute bronchitis 2. Acute respiratory tract infections 3. Chronic asthma 4. Chronic obstructive pulmonary disease (COPD) 5. Sore throat |
| **Diseases of the digestive system**   1. Peptic acid disease | **Diseases of the digestive system**   1. Abdominal pain in ulcerative colitis 2. Crohn's disease 3. Functional constipation 4. Functional dyspepsia 5. Inflammatory bowel disease 6. Irritable bowel syndrome (IBS) 7. Symptomatic gastroparesis 8. Small bowel obstruction (SBO) 9. Ulcerative Colitis (implied in "abdominal pain in ulcerative colitis") |
| **Diseases of the skin**  NA | **Diseases of the skin**   1. Atopic dermatitis 2. Atopic eczema |
| **Diseases of the musculoskeletal system or connective tissue**   1. Osteoarthritis | **Diseases of the musculoskeletal system or connective tissue**   1. Back pain 2. Chronic nonspecific low back pain 3. Fibromyalgia 4. Hip osteoarthritis 5. Knee osteoarthritis 6. Low back pain (LBP) 7. Musculoskeletal pain 8. Neck pain 9. Osteoarthritis 10. Rheumatoid arthritis 11. Temporomandibular disorders |
| **Diseases of the genitourinary system**  NA | **Diseases of the genitourinary system**   1. Chronic prostatitis and chronic pelvic pain syndrome (CP and CPPS) 2. Chronic prostatitis 3. Endometriosis 4. Nephrotic syndrome 5. Polycystic ovarian syndrome 6. Subfertile women with polycystic ovarian syndrome 7. Urinary incontinence in women 8. Chronic pelvic pain |
| **Pregnancy, childbirth or the puerperium**  NA | **Pregnancy, childbirth or the puerperium**   1. Breast engorgement during lactation 2. Induction of labour 3. Labour pain 4. Miscarriage 5. Pregnancy pain 6. Preterm and/or low birth‐weight infants 7. Threatened miscarriage 8. Unexplained recurrent miscarriage 9. Breast milk production (Lactation) |
| **Certain conditions originating in the perinatal period**  NA | **Certain conditions originating in the perinatal period**   1. Procedural pain in newborn infants |
| **Symptoms, signs or clinical findings, not elsewhere classified**   1. Pain 2. Nausea 3. Fever 4. Inflammation | **Symptoms, signs or clinical findings, not elsewhere classified**   1. Abdominal pain 2. Chronic fatigue 3. Pain 4. Skeletal muscle cramps 5. Stress |
| **Injury, poisoning or certain other consequences of external causes**   1. Severe injury | **Injury, poisoning or certain other consequences of external causes**   1. Falls in Parkinson's disease 2. Postoperative dental pain 3. Postoperative nausea and vomiting 4. Postoperative urinary retention |
| **Factors influencing health status or contact with health services**  NA | **Factors influencing health status or contact with health services**   1. Pelvic floor muscle training |

Note: NA, not available.

**Reference**

World Healt Organization. (2002). WHO traditional medicine strategy 2002-2005. Geneva, Switzerland, 74, Avaialble at: <https://www.who.int/publications/i/item/WHO-EDM-TRM-2002.1>. Accessed on: October 13, 2025.

World Healt Organization. (2024). International Classification of Diseases 11th Revision. The global standard for diagnostic health information, Geneva, Avaialble at: <https://www.who.int/standards/classifications/classification-of-diseases>. Accessed on: October 13, 2025.
